# Novel Plant Breeding Techniques Shake Hands with Cereals to Increase Production

**DOI:** 10.3390/plants11081052

**Published:** 2022-04-12

**Authors:** Muhammad Haroon, Xiukang Wang, Rabail Afzal, Muhammad Mubashar Zafar, Fahad Idrees, Maria Batool, Abdul Saboor Khan, Muhammad Imran

**Affiliations:** 1 National Key Laboratory of Crop Genetic Improvement, Huazhong Agricultural University, Wuhan 430070, China; rabail.afzal.10@gmail.com; 2 College of Life Sciences, Yan’an University, Yan’an 716000, China; 3 State Key Laboratory of Cotton Biology, Key Laboratory of Biological and Genetic Breeding of Cotton, Chinese Academy of Agricultural Science, Anyang 455000, China; m.mubasharzafar@gmail.com; 4 College of Plant Science and Technology, Huazhong Agricultural University, Wuhan 430070, China; fahad.idrees332@gmail.com (F.I.); maria.batool@webmail.hzau.edu.cn (M.B.); 5 Institute of Plant Sciences, University of Cologne, 50667 Cologne, Germany; akhan7@smail.uni-koeln.de; 6 State Key Laboratory for Conservation and Utilization of Subtropical Agro-Bioresources, College of Agriculture, South China Agriculture University, Guangzhou 510642, China; imran_m1303@yahoo.com

**Keywords:** plant breeding, genome editing, molecular breeding, prime editing, base editing, CRISPR Cas, epigenetics, speed breeding

## Abstract

Cereals are the main source of human food on our planet. The ever-increasing food demand, continuously changing environment, and diseases of cereal crops have made adequate production a challenging task for feeding the ever-increasing population. Plant breeders are striving their hardest to increase production by manipulating conventional breeding methods based on the biology of plants, either self-pollinating or cross-pollinating. However, traditional approaches take a decade, space, and inputs in order to make crosses and release improved varieties. Recent advancements in genome editing tools (GETs) have increased the possibility of precise and rapid genome editing. New GETs such as CRISPR/Cas9, CRISPR/Cpf1, prime editing, base editing, dCas9 epigenetic modification, and several other transgene-free genome editing approaches are available to fill the lacuna of selection cycles and limited genetic diversity. Over the last few years, these technologies have led to revolutionary developments and researchers have quickly attained remarkable achievements. However, GETs are associated with various bottlenecks that prevent the scaling development of new varieties that can be dealt with by integrating the GETs with the improved conventional breeding methods such as speed breeding, which would take plant breeding to the next level. In this review, we have summarized all these traditional, molecular, and integrated approaches to speed up the breeding procedure of cereals.

## 1. Introduction

With the burgeoning human population, the demand for food has put a lot of pressure on our agricultural system [1]. It is estimated that in the next 50 years, the agricultural system needs to double up the production of food to feed more than nine billion people [2,3]. In human feed, cereal crops are known as the staple food contributing approximately 66% of the food supply worldwide. The rise in population will require a 38–67% increase in cereals’ production, i.e., rice, maize, and wheat [4,5].

**Figure 1:**
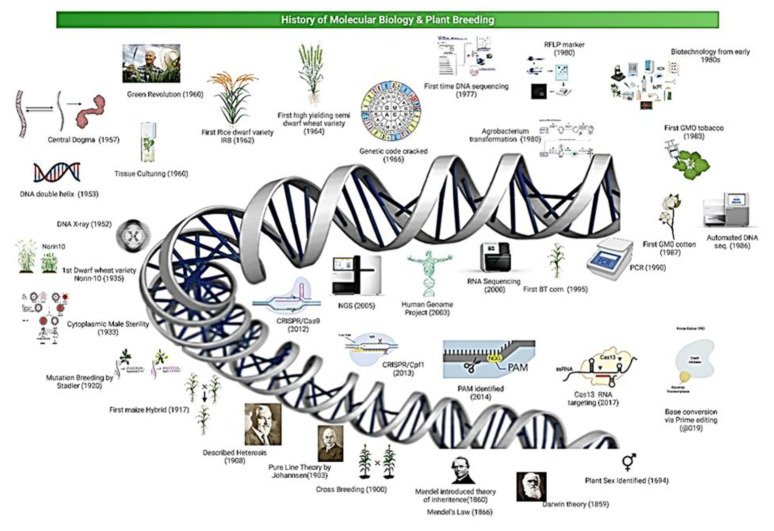
I History of molecular biology and plant breeding with major events that happened with the passage of time.

Since the start of the 1940s, the varieties developed by Norman Borlaug have focused on developing high-yielding and disease-resistant wheat varieties; Mexico became the first country to start the export of wheat in the 1960s [6]. During the green revolution era, agricultural production was boosted from 1 billion tons in 1960 to 2 billion tons in 2000 [7]. However, agricultural researchers are still using those conventional breeding methods, but it takes 6–8 years to develop a new variety [8,9] Additionally, limited allelic diversity in the crops’ genome hampers the breeding process to improve cereals’ genome in a shorter time. These issues have arisen due to the urge for another revolution to meet the increasing food demand [8,9]. In keeping with the currently prevailing problems, the plant breeding paradigm has shifted and now aims to combine both conventional and novel GETs to increase the production of cereal crops in a short period by reducing the selection cycles and increasing the genetic diversity [6,10]. In conventional GETs, site-specific nucleases (SSNs) which have DNA binding domains or RNA sequences have been used to target and edit the genome. Various SSNs such as meganucleases, transcription activator-like effector nucleases (TALENs), and zinc-finger nucleases (ZFNs) have become outdated due to their dependency on few loci, construction of specific enzymes, high cost of specific protein domain construction, suitable vector construction with specific monomers, and associated off-target effects [11]. However, with the advancement in molecular biology and plant breeding (Figure 1), new and novel GETs (CRISPR/Cas9, CRISPR/Cpf1, prime editing, base editing, epigenetic modification tool) have been developed to edit plants’ genomes precisely and efficiently in less time (Table 1) [12].

CRISPR/Cas9 and CRISPR/Cpf1 use the double-stranded DNA breaks (DSBs) at the targeted sites to cleave the DNA and insert new genes. The repairing machinery activates and repairs the cleavage sites by non-homolog end joining (NHEJ) or homology-directed repair (HDR) [13]. Moreover, few GETs work independently without inducing the double-stranded DNA breaks (DSBs), including prime editing, base editing, and dCas9-based epigenetic modification. These GETs can also be associated with unwanted DNA mutations. Furthermore, transgenic events in these crops are regulated and approved by the Food and Drug Administration (FDA), Environmental Protection Agency (EPA), and United States Department of Agriculture Animal and Plant Health Inspection Service (USDA/APHIS) before they are released to farmers for crop production [14]. The world population is rising at a very high pace and the conventional breeding methodologies take almost 8–12 years to develop an improved variety and so will not be able to keep pace with changing climatic conditions and rising food demands. Therefore, newly developed varieties, which can cope with the changing climatic conditions without comprising the yield potential, are needed in a much shorter time. 

The novel GETs such as prime editing, base editing, and dCas9-based epigenetic modification [15] can be coupled with an advanced breeding method called “speed breeding” [16]. Speed breeding speeds up the breeding procedure by producing six generations per year in wheat [17]. Despite the development of novel GETs, it still requires a tissue culturing process, which is complicated and time-consuming. Therefore, the newly developed method, “ExpressEdit”, amalgamated with speed breeding, can exclude the complex callus formation [18]. Through the speed breeding process, aided with GETs, yellow rust, stripe rust, and many other diseases have been reduced in spring wheat by producing 4–6 generations in a year [16]. In this review, new GETs, conventional breeding methods, and the integration of conventional and GETs methods are discussed. Moreover, the applications of these approaches and future prospects to increase cereals’ production are also elaborated on at length. 

## 2. Development of Genomic Technologies and Role in Plant Breeding

The innovation of NGS technology has opened new ways to decipher the genome complexity for the improvement of crops [34,35]. Furthermore, genome-wide molecular tools (several molecular markers, high-density genetic maps, genotyping strategies, etc.) have also played their role in selecting the best candidate lines and improving plants’ genomes [13,14,36]. Recent genomic innovations have accelerated the breeding procedures by modifying the selection methods that are responsible for screening a large amount of data with more precision and efficient breeding (marker-assisted selection, association mapping, breeding by design, genomic selection, gene pyramiding, etc.) [37,38,39]. NGS is 1000 times cheaper than Sanger sequencing, which generates a vast array of genomic information [40]. Furthermore, after the completion of the human genome project and rice genome sequencing, the costs of NGS declined and have continued to decline [41,42]. NGS platforms have achieved marvelous achievements in different plant science fields such as plant breeding, agri-genomics, and functional genomics [43]. Despite the potential uses of NGS in agriculture, it still has to pass through several challenges such as the generation of short sequence reads (35–700), which are not considered efficient in the case of the complex, big, and repetitive genome, and the detection of rate mutation in a plant’s genome [44,45]. However, to overcome the bottlenecks such as NGS inefficacy and mutation detection, Stahlberg et al. and Monson-Miller demonstrated the use of Multiplexed, PCR-based bar-coding of DNA for selective mutation detection using sequencing (SiMSen-Seq) and Restriction Enzyme Sequence Comparative Analysis (RESCAN) [45,46]. The innovations in NGS have conclusively provided us with genome sequences of several crops that will facilitate the more efficient and precise use of GETs. 

## 3. Genome Editing with DSBs

### 3.1. CRISPR from Yogurt to Plant Breeding

**Figure 2:**
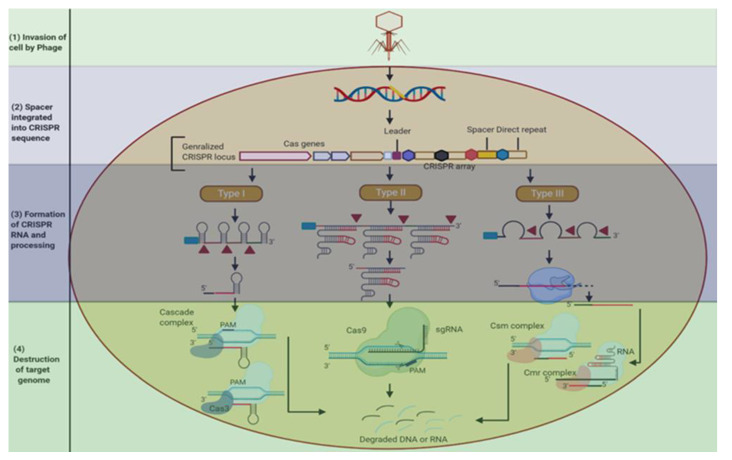
Schematic mechanism of bacterial CRISPR system as a defensive tool to degrade the viral genome. Step 1: During the invasion, foreign genetic material (viral genome) enters in bacterial genome. Step 2: (Integration of spacers); spacers are inserted into the genome (shown in yellow color) and this is memorized by bacteria to recognize in case of future invasion. Step 3: (CRISPR-RNA formation and processing); CRISPR array is a noncoding part that is maturated during this step and works only according to a specific CRISPR system mentioned in figure. In CRISPR type I and III, associated ribonucleases in CRISPR work to cleave the pre crRNA between the repeats and liberate many short crRNAs. System III-associated crRNA goes through a further process at 3′end by employing the RNases that are yet to be identified and produce maturated RNA transcript. Step 4: (Destruction of target genome); for the recognition and destruction of the target sites, type I and III have several complexes of proteins with crRNAs. The cascade complex is present in type I, and Csm and Cmr complexes are present in type III for DNA and RNA cleavage, respectively. The cas3 nuclease bounded with the R-loop facilitates the process in type I, whereas type II has fewer proteins and cas9 is required for degradation. Protospacer adjacent motifs (PAMs) in type II facilitate the cas9 in identifying the target sites. In both I and II types, self-targeting of CRISPR is prevented due to the lack of PAM in the targeted sequences.

In 1987, the bacterial genome was sequenced to study the defensive mechanism and found repetitive sequences in the genome, which in 2005 were named as CRISPRs. Furthermore, it was found that the viruses that attack bacteria share some similarities with the sequences present in the bacteria. The matched CRISPR sequences were later confirmed by Danisco while studying the defensive mechanism of yogurt bacteria that survived against the viral attack [47]. The CRISPR mechanism was then studied in detail and the Cas genes associated with it were found. CRISPR loci are surrounded by different *Cas* genes and repetitive sequences, and are interspaced by variable sequences (spacers), which correspond to the sequences present in foreign genetic elements called protospacers. *Cas* genes translate themselves into proteins and degrade the genome of foreign genetic elements such as viruses [48]. The Cas genes were also identified as having the ability to cut the DNA by encoding domains of proteins as explained in [49,50]. These associated genes serve as the basis for classifying CRISPR into three different types (I, II, III) (Figure 2) [51]. Each of these three types are distinguished by the presence of specific genes: Cas3 gene in type I; Cas9 gene in type II; Cas10 in type III. Type I and III have different Cas proteins that also form complexes with crRNA (CRISPR-RNA) to assist the target nucleic acids’ identification and destruction [52]. Type II has a smaller number of Cas proteins and their biological importance is still elusive [53]. Moreover, type II is the most commonly used due to the high accuracy in cutting and generating DNA and crRNA, respectively. It consists of two domains, RuvC and HNH, that are responsible for the DSBs of targeted DNA, hence making this type more precise and carrying out genetic engineering at a very low cost [54]. The CRISPR/Cas system has widely shown its role in all living organisms. The human genome has also been edited using CRISPR technology to knock out genetic diseases. Recently, CRISPR has been used to study the viral infection COVID-19. The high sensitivity and specificity of CRISPR has the ability to detect variation in even a single nucleotide, which leads this system to be considered as more reliable and efficient in detecting viral diseases in humans. Moreover, CRISPR has been considered as a major advancement in plant improvement either by improving crop yield, resistance to biotic and abiotic stresses, or diversity in plant species [55,56]. 

### 3.2. CRISPR/Cas9 and Cpf1 in Genome Editing

The development of the CRISPR/Cas9 mechanism (Figure 2) for the improvement of crops is based on the bacterial defensive mechanism. While CRISPR/Cas9 functioning is performed in three steps: (1) Acquisition: acquisition of spacer DNA from the viral DNA or resident plasmids is required due to the presence of DSBs, which results in insertion in the bacterial genome (to memorize the invading viral DNA); (2) Expression: expression of crRNA from the transcription of the CRISPR array, which also involves the expression of the Cas9 protein; (3) Interference: crRNA acts as a guide RNA, which is further directed by Cas9 protein to bind at targeted DNA that is accompanied by PAM sites and cuts the specified DNA three-nucleotide away from PAM sites at both DNA strands [57]. 

In plants, the CRISPR/Cas9 system edits plants’ genomes by employing various components, including Cas9 protein and sgRNA. Initially, sgRNA is designed in silico, which is an amalgamation of crRNA and tracrRNA. However, thanks to bioinformaticians, many online algorithm-based software and websites are available to design the very specific and precise sgRNA, for example, CRISPR-P, CHOPCHOP, etc. [2,40]. It is compulsory to construct both expression cassettes of Cas9 and sgRNA separately. Small nuclear RNA gene promoters U3 or U6 are used for the transcription of sgRNA by using RNA polymerase 3 and defining the initiation and termination site. 

For a successful cleavage of specified sites, sgRNA and targeted DNA sequences should be matched, except for the first nucleotide (5′G or A). During the Cas9 expression and its nuclear localization purpose, single or dual NLS (nucleic localization signal) is fused with the Cas9 coding sequence (4107-bp). Both Cas9 and sgRNA expression cassettes are assembled in vectors to perform further genome editing procedures. Before conducting a final genome editing step, protoplasts are transformed with the CRISPR to analyze and validate the sgRNA activity [58]. Next, a PCR or restriction enzyme digestion step is employed to select the active CRISPR. The final vector contains the CRISPR/Cas9 setup, which is transformed in the plant cells via *Agrobacterium*-mediated transformation or a particle bombardment procedure [59]. After transformation in a plant cell, the following steps are carried out: the activation of Cas9 proteins, cleavage at targeted sites, and production of DSBs. The activation step involves the gRNA activating the Cas9 protein. Without the binding of gRNA, Cas9 protein is nonfunctional. Bacteria (*Streptococcus pyrogens*) have a protein named Cas9 (originally called SpCas9), which is widely used in plants and has the uniqueness to recognize the NGG type PAM site. 

**Figure 3:**
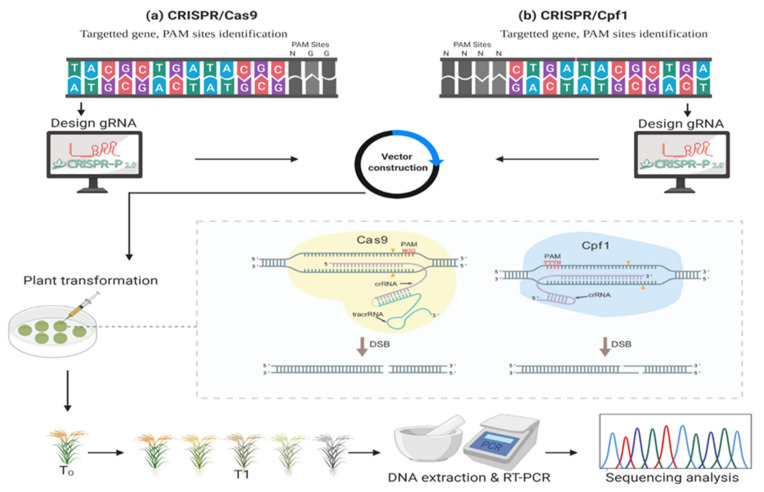
Mechanism of CRISPR/Cas9 and Cpf1 to edit the plant’s genome; (**a**) is a schematic view of CRISPR/Cas9, and (**b**) is of CRISPR/Cpf1. Both GETs are used to edit the plant’s genome. In both GETs, initially, desired DNA and PAM (20 sequences) sites are selected in plants’ genome. Different sgRNA designing bioinformatics tools are available, which gives information about the best gRNA for subsequent GE steps. sgRNA is cloned and vector is constructed to deliver in the genome by using *Agrobacterium tumefaciens*-mediated plant transformation. By using a couple of steps, transgenic plants are developed (shown in dotted line box). Further transgenic plants are regenerated and screened by genotyping analysis.

The CRISPR/Cas9 technique (Figure 3) is being continuously improved for efficient genome editing. CRISPR is categorized into two classes based on the effector molecules they have: class 1 and class 2. Class 1 contains multiple subunits of effector molecules containing different Cas proteins, while class 2 contains a single effector protein [60]. Furthermore, these classes are divided into six subtypes; I, II, III, IV, V, and VI. Class 1 contains type I, III, and IV, while class 2 has type II, V, and VI. These types contain different Cas genes; Cas3 in type I, Cas9 in type II, Cas10 in type III, type IV is a putative subtype, Cas12 in type V, and Cas13 in type VI [61]. Among these types, type II is the most commonly used due to its high efficiency in genome editing. Although class 1 accounts for 90% of the CRISPR/Cas system, it is less studied and rarely used in genome editing due to its complex system [62], while class 2 is more abundantly studied and used in genome editing due to the presence of Cas9, Cas12, and Cas13 genes. 

Recently, the type V CRISPR/Cas system has been identified with several subtypes. The main studied types are Cpf1 (Cas12a) as type V-A and C2c1 (Cas12b) as type V-B. Cpf1 is now considered a better substitute for Cas9 due to its efficient version of GETs. CRISPR/Cpf1 (Cas12a) refers to CRISPR from *Prevotella* spp. and *Francisella* spp. Furthermore, CRISPR/Cpf1 has been adapted more than CRISPR/Cas9 due to its short sgRNA nucleotide length and reduced size of the Cpf1 protein. Its sgRNA only requires shorter crRNA as compared to both crRNA and tracrRNA in the CRISPR/Cas9 mechanism [63,64]. The sgRNA directs the Cpf1 nuclease to bind at the targeted region upstream of PAM. In comparison to Cas9 protein, Cpf1 prefers T-rich PAMs instead of G and cleaves the targeted DNA at the proximal site of PAM in a staggered fashion to generate blunt ends [65]. CRISPR/Cpf1 has been used in many plants [66]. Furthermore, it is necessary to insert or delete the nucleotide sequences for the improvement of crop traits. For this purpose, the natural repairing mechanism of cell machinery is switched on. Generally, HDR and NHEJ nucleotide repairing mechanisms work to insert the nucleotide sequences precisely at the cleavage site or random insertion or deletions [67].

Recently, CRISPR/Cas12b (C2c1) has been developed, which is a dual RNA-guided endonuclease similar to Cpf1. Cas12b has the ability of temperature inducibility; hence, it can be used for developing plants’ resistance to high temperatures. Cas12b has the longest sticky ends of all the CRISPR systems, producing DNA DSBS with 6–8 nucleotide sticky ends. The size of Cas12b is smaller than Cas9 and Cas12a. Moreover, Cas12b, just like Cas9, needs a crRNA and tracrRNA combined with an sgRNA for DNA targeting [68]. 

### 3.3. Genome Editing (with DSBs) Role in Cereal’s Genome Improvement

To date, the GETs such as CRISPR/Cas9 and Cpf1 have been used to increase the production and disease resistance of crops as shown in Table 2. CRISPR/Cas9- and Cpf1-based GETs are more efficient than endonucleases/meganucleases (EMNs), meganucleases (MNs), ZFNs, and TALENS, which were a breakthrough in the agricultural arena to improve plants’ targeted traits with more precision, accuracy, and minimized off-target effects [2,47,48]. These GETs are very broad to be applicable for the improvement of cereal crops [69,70,71]. 

### 3.4. Genome Editing without DSBs and Donor Template

**Figure 4:**
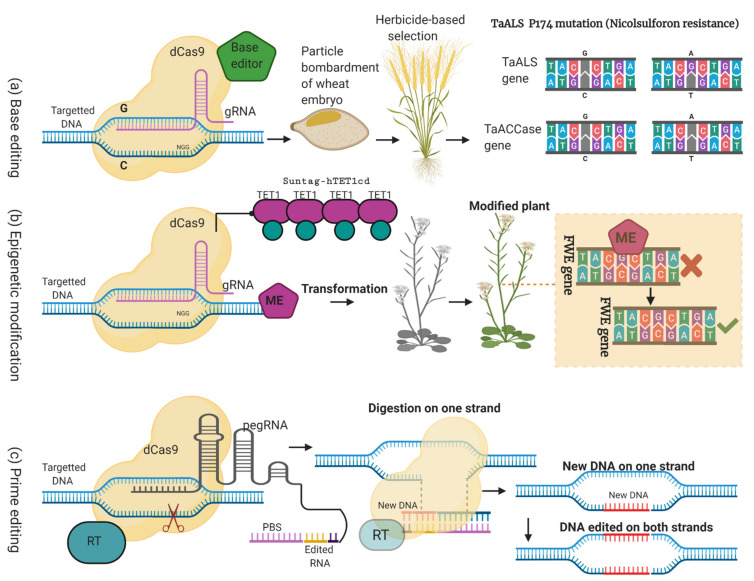
A modified form of a figure from [107,108], which shows the novel GETs without producing the DSBs; (base editing (**a**), epigenetic modification (**b**), and prime editing (**c**)). In (**a**), by using the base editing approach, two genes (*TaALS* and *TaACCase*) are co-edited. This approach is used by coupling the dCas9 with a cytosine base editor (CBE). In this way, such types of transgenic wheat plants are developed, which did not produce any DSBs. (**b**) is epigenetic editing; in this approach, dCas9-Suntag-hTET1cd is coupled with dCas9 for demethylation of the FWA promoter to activate the FWA gene expression. (**c**) is prime editing that works by developing a complex interaction between pegRNA, Cas9 nickase-reverse transcriptase (RT), and target DNA. In the pegRNA, except for the primer binding site (PBS), the desired genome sequence is also present, which is introduced in the host genome. For RT, pegRNA produces primer; RT copies the information of pegRNA, and the RT product is integrated with the target genomic site. Initially, modification happens only at one targeted DNA strand. Later, modification is present on both strands due to the cell’s repairing mechanism.

CRISPR/Cas9 is a versatile tool used to edit the plant’s genome precisely and with efficacy. Despite its countless services for the betterment of the plant’s genome, it may cause harmful mutations owing to off-target effects. These mutations may leave unpredictable results in the next generations. There are ways to detect these off-target mutations either in vitro or in vivo such as CIRCLE-seq, GUIDE-seq, DISCOVER-seq, SITE-seq, and Digenome-seq [129]. These mutations are caused by DSBs. However, to cope with the off-target mutations, brave approaches can be used without inducing the DSBs (Figure 4) [107,108] to insert the genome at the targeted DNA [130]. 

New approaches such as base editing [131] and prime editing [132] exploit the Asp10Ala and His840Ala mutations containing the dCas9 protein with other effector proteins to bind at specified genome locations. This dCas9 protein can alter the single base pair without any cleavage in that region [133]. It has no more nuclease activity but works to guide the sgRNA for binding.

#### 3.4.1. Base Editing

Genome editing requires gRNA, the Cas9 protein, donor template, and repairing mechanism for the editing of the genome, while base editing uses the reprogrammable deaminase intending to introduce the bases at the targeted sites without any cleavage and induction of DSBs [134]. For this purpose, the CBE (cytosine base editor) and ABE (adenine base editor) have been developed to alter the C-T and A-G, respectively [69]. In humans, daily spontaneous hydrolytic deamination causes the conversion of C-T and A-G 500 times per cell [135]. ABE contains different base editors, including Target-AID and BE. In Target-AID, the pmCDA protein is fused with the dCas9 protein (Cas9n, D10A) to perform base editing. In BE series, the rAPOBEC protein is used for fusing with the dCas9 protein (Cas9n, D10A). CBE is used to alter the C-T, and then T is changed to U in response to the natural repairing mechanism. The CBE genome editing technique has already been used in crops including, tomato, wheat, rice, maize, and *Arabidopsis*, while, ABE is used to deaminase A to G, and has been reported in wheat, rice, *Arabidopsis*, and *Brassica napus* [69]. Its improvement of cereals’ genomes has been discussed in the section “Genome Editing (Without DSBs) Role in Cereals’ Improvement”.

#### 3.4.2. Epigenetic Editing 

Epigenetic refers to the modification of the genome without perturbing the DNA sequences such as histone modification, DNA methylation, DNA demethylation, gene imprinting, chromatin remodeling, etc. [136]. These epigenetic modifications are common in plants [137]. Nature has blessed plants with a specialized mechanism of epigenome editing to protect against various kinds of biotic and abiotic stresses [138]. CRISPR/Cas’s component Cas9 protein is exploited in the form of dCas9 for the genome modification. Protein dCas9 is fused with the epigenetic modifier for the targeted modification, which results in the alteration of gene expression [133]. For example, Gallego-Bartolomé and his colleague worked to modify the plant’s genome epigenetically by involving DNA demethylation/methylation resulting in targeted DNA methylation, and a late flowering phenotype was developed [133]. These epigenetic modifications are also maintained in the next segregates. However, a lot of work is needed to explore this technology in all other cereals.

#### 3.4.3. Prime Editing

Prime editing is also a new genome editing technique that utilizes the Cas9 nickase amalgamated with a PE guide RNA (pegRNA) to edit the genome precisely by a “search and replace mechanism” [139]. In the CRISPR/Cas9 mechanism, DSBs are generated that are associated with some complex off-target effects, including p53 activation and translocations [140]. Prime editing technology was developed first by Liu and his colleagues in 2019 [141]. This technique can perform insertions, deletions, and all base conversions without requiring a donor template and the production of DSBs. The prime editing system is a combined work using the Cas9 nickase fusion protein, engineered reverse transcriptase enzyme, and pegRNA. This programmable pegRNA is designed to carry the information about the binding sites and replace targeted DNA nucleotides with the desired genetic information [139]. The main objective was to increase the efficiency of genome editing. For this purpose, three main developments were achieved, including prime editor 1, prime editor 2, and prime editor 3. In plants, prime editing has been successfully employed in wheat, rice, and maize [142]. More research is needed in plants to make this technology capable of being used for many nucleotide insertions or deletions without creating DSBs. However, for a small number of nucleotide insertions and deletions, it is considered more efficient than the CRISPR/Cas9 gene editing tool [139]. The advancement in prime editing has developed an improved system called engineered plant prime editor (ePPE). The efficiency of pegRNA has been enhanced by combining it with ePPE. Recent research on ePPE has reported the development of rice plants tolerant to herbicides such as sulfonylurea and imidazolinone [142]. 

## 4. Genome Editing (without DSBs) Role in Cereals’ Improvement

All genome editing techniques have pros and cons. Similarly, CRISPR/Cas9 and other GETs such as MNs, ZFNs, and TALENs are associated with off-targeted mutations, low efficiency, and PAM sites dependency. These limitations can be addressed by using the newly developed methods, such as base editing and prime editing tools, which do not require any DSBs for altering plants’ genomes and can further alleviate the GMO’s concerns [15]. 

A base editing tool was initially reported in rice by employing the rat cytidine deaminase enzyme “APOBEC1”, which is fused to the N-terminus of the Cas9 nickase protein. As a result of the fusion with APOBEC1, the Cas9 nickase becomes programmable with the association of gRNA [143]. By using the base editing tool, two rice genes (*NRT1.1B* and *SLR1)* were edited to increase the nitrogen use efficiency and reduce height, respectively [144,145]. The same tool was used to induce the point mutation in rice to check the vector’s performance and feasibility. Several studies reported plants containing the sgRNA-APOBEC1-XTEN-nCas9-UGI vector, and its efficacy was checked on three targets: (P2) in the *OsPDS* and (S3 and S5) in the *OsSBEIIb,* which encodes phytoene desaturase and starch branching enzyme IIb in rice, respectively. Rice calli were transfected by using the *Agrobacterium*-mediated transformation protocol. Furthermore, a targeted sequence can determine the efficiency of vectors up to 20% [146]. According to our understanding, various groups have used the base editing tool to improve plants’ genomes [147]. 

From a historical perspective, prime editing was initially developed to make DNA-free gene editing in mammals and yeast [139]. Later, this system was modified to develop the prime editor 2 (pPE2) system for genome editing in rice. Base editors have also played a profound role in improving the important agronomic traits, including plant height, disease resistance, and flowering time. However, base editors could not meet the challenges such as base transversions and insertions in plants’ genomes, while prime editing addressed the already existing problems in base editors to increase the efficiency of transgenic plants [132]. Wei et al. employed a pPE2 system to induce the point mutation at different targeted sites of the rice genome. Different genomic sites depicted varying mutation frequency rates (0–31.3%) [132]. Furthermore, prime editing tools are being used to edit plants’ genomes with increased efficiency and efficacy. However, a lot of research is needed to make this technology widely available in the agricultural field to improve the important agronomic traits of different crops.

Epigenetic modifications are inheritable in the next generation without GMO regulatory concern. Naturally, epigenetic modifications happen in plants’ genomes, which are considered to be non-GMO crops. The CRISPR/Cas9 component, the Cas9 protein, was modified to the dCas9 protein to alter plants’ genomes epigenetically, as with DNA methylation, which can perform gene silencing, and many other epigenetic modifications [69]. Protein dCas9 and different modifiers can be fused to improve the important traits. For the first time, a dCas9 system (dCas9-SunTag-TET1) was fused with the human DNA demethylase (TET1cd) to target the *FWA* gene and upregulated the expression of the *FWA* gene, which demonstrated the late flowering of plants in *Arabidopsis* [148]. However, the same tool can be employed in other cereal crops to cause DNA methylation for modifying plants’ genomes in pursuance of beneficial agronomic traits without any GMO regulatory objections.

## 5. Developing Genome-Edited Plants Free of Transgene

The transformation of plants is usually achieved by a tissue culturing technique, which is time-consuming, costly, and intensive [149]. Another big hurdle in genome editing is the chance of off-target mutations [150]. Therefore, transgene-free plants are more preferred to minimize the effects of off-target mutations. The *Cas9* gene is undesirable in this regard as it often causes the induction of off-target mutations [151].

**Figure 5:**
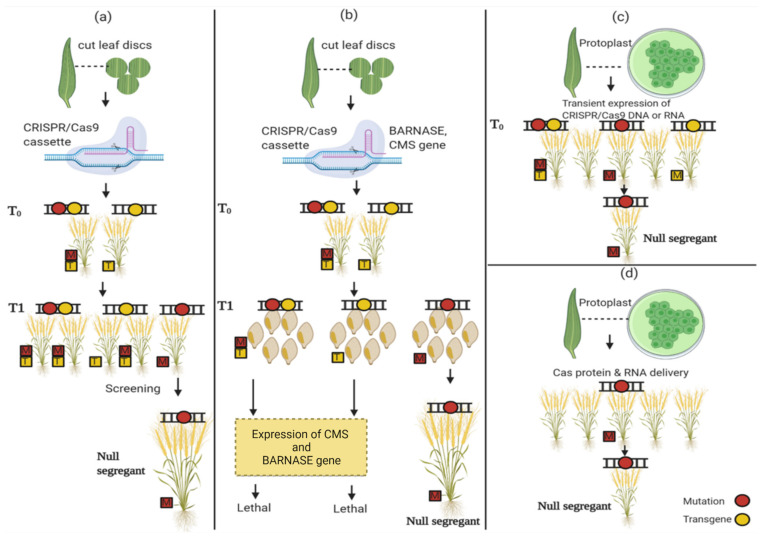
The figure illustrates the variety of development processes without transgenes by using CRISPR/Cas9. In these methods, null segregants are produced and isolated by using the Mendelian segregation; (**a**) Isolate segregants by Mendelian segregation, (**b**) programmed self-elimination of transgene plants, (**c**) transient CRISPR/Cas9 gene expression by protoplast, (**d**) RNP-mediated genome editing. For the detailed study, please refer to the Section 5.1 below.

Some countries require the removal or alleviation of transgenes to ease their regulatory concerns over GMOs. Based on these concerns, researchers are trying to develop null segregants and achieve this targeted mutation through RNPs (ribonucleoproteins), which is the most efficient strategy (Figure 5) [47,129]. Another approach that can be used for this purpose is the selection and regeneration of mutant plants without any pressure of precise selection [152]. However, this approach to obtain transgene-free plants is time-consuming and laborious with a low-efficiency rate [69]. Some other approaches that can be used for this purpose are discussed below.

### 5.1. Isolate Segregants by Mendelian Segregation

This is the most common approach for obtaining transgene-free plants by isolating the null segregants through the Mendelian approach. This involves the selection of plants based on antibiotic resistance by using the CRISPR/Cas9 gene cassette. In this approach, after the identification of the genome-edited plants, the plants are grown to obtain progenies [153]. The transgenes are segregated in the progenies according to the law of segregation. Thus, the selected genome-edited plants have high chances of fewer or no transgenes. The screening can be performed more efficiently by using PCR and other technologies to identify the Cas9 free plants. However, this approach is time-consuming and laborious [152]. 

### 5.2. Programmed Self-Elimination of Transgene Plants

In this approach, two suicide genes, *BARNASE* and *CMS,* are placed in the control of the REG2 and 35S promoter, respectively, to isolate the null segregants [154]. The *BARNASE* gene is a toxic protein having the ability to kill plant cells. Embryos that contain transgenes are killed by the *BARNASE* gene. The *CMS2* gene affects the mitochondrial functions and causes male sterility [155]. This approach can be used to isolate the null segregants but does not work on asexually propagated plants. [156]. 

### 5.3. Transient CRISPR/Cas9 Gene Expression by Protoplast

Another method to obtain the null segregant can be achieved by using DNA or mRNA by the transient expression of CRISPR/Cas9. In 2017 and 2018, Anderson and Lin, respectively, isolated the protoplast from potato and nine other plant species [157,158]. In both studies, mutagenesis was achieved, and the vector sequences were observed in 10% of the potato lines and 17.2% of the *Nicotiana tabacum* genome-edited lines. The problem with using the protoplast method is the specificity of the protocol for each species and limited plants’ regeneration. Both studies indicated the usefulness of this method for the isolation of segregants and plants regenerated did not show any transgene. However, the risk of transgene integration always persists. Thus, this method needs screening of the plants to be more efficient and precise [159]. 

### 5.4. RNP-Mediated Genome Editing

Genome editing using RNA is the most reliable method being used for transgene-free plants. The RNA can be transferred to the protoplast through in vitro culture. Due to DNA-free RNA-transformation, the transgene-free plants can be produced [160]. Woo was the first researcher who demonstrated the CRISPR/Cas9 delivery in protoplasts. The researchers using this methodology obtained results with an efficiency ranging from 8.4% to 44% [161]. With time, the successful transformation of RNP-mediated genome editing was reported in fruits (apples, grapevine) and cereals (wheat, maize, rice). The approach was proven to succeed with an efficiency rate ranging from 4 to 64% without selection [87]. The efficiency in maize can be increased from 2.4 to 47% using selection markers. A considerable reduction in mutations in off-target regions has been observed by using this approach [162]. 

## 6. Speed Breeding 

**Figure 6:**
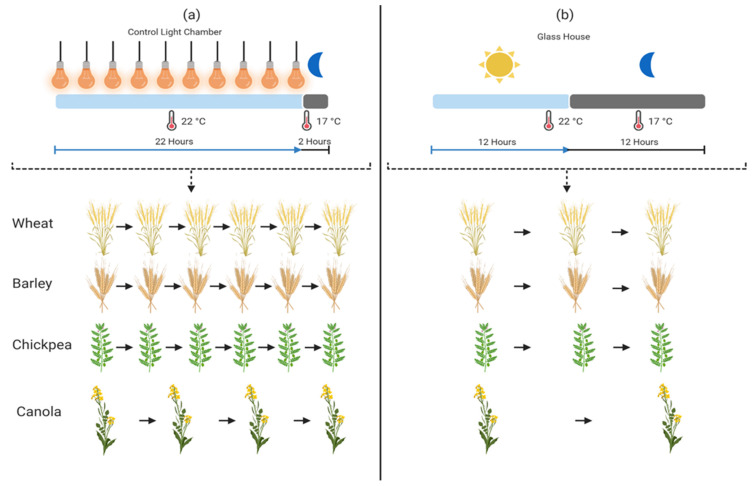
Speed breeding has taken plant breeding to the next level by achieving 5–6 generations in a year. Speed breeding chamber (22 h artificial light, 2 h dark) as shown in part (**a**) as compared to glasshouse (12 h sunlight, 12 h dark) (**b**), has produced 6 generations of wheat, barley, chickpea, and 4 generations of canola, while glasshouse (**b**) can only produce 3 generations of wheat, barley, canola, and 2 generations of canola. For a further detailed explanation of the performed research, refer to Section 6 on speed breeding.

Breeding by conventional breeding methods has shown significant results for many years. Numerous varieties and lines have already been developed by using the conventional breeding methods. However, with the passage of time, increasing food demand is outstripping the current production levels; hence, the dissemination of improved cultivars is needed with a substantial increase in production efficiency and abiotic and biotic stress tolerance [163]. Moreover, with the changing climatic conditions, crop production has experienced a significant decrease, which builds pressure on researchers to use suitable techniques for crop improvements [164]. Developing varieties with conventional methods takes a lot of time usually: 8–10 years from first cross to variety release [165,166]. This pushed scientists to develop a new methodology to hasten breeding procedures by reducing the time required to develop new lines, which led to the introduction of a new technique called “speed breeding” [167]. Speed breeding speeds up the breeding programs with its unique technique by increasing the number of generations per year (Figure 6) [16]. This method was first used in wheat by NASA in space by extending the photoperiod with optimum temperature [168]. This method was then improved by the scientists of Queensland University by bringing it to the earth and utilizing it in their greenhouses. This was then named speed breeding and was first practiced on wheat with the purpose of decreasing the generational period. Thus, the NASA-inspired method to enhance the genetic gain has been reported to boost wheat production by up to three times [10]. Speed breeding is a flexible procedure that works in different ways. The simplest method is to increase the continuous lighting in greenhouses, which allows plants to grow faster, and the increased photosynthesis rate boosts growth and development [166,169]. Plants have adapted according to the area where they grow, and the need for light intensity and duration is developed with the adaptation. Hence, they are referred to as long-day and short-day plants considering the light that plants need [170]. The continuous lighting in long-day plants accelerates the reproductive cycle, thus reducing the vegetative phase and allowing researchers to obtain more generations per year, while the short-day plants require a particular lightening scheme to reach the reproductive phase in a short period of time [171]. The protocol for this technique is different for each crop relative mainly to their day length requirement, which directly affects their flowering time period [170]. With the adjustment in light intensity and duration, the speed breeding technique also considers other factors of plant development such as temperature requirement, humidity, and soil composition, which change with each crop [172]. The source of light is the key factor in speed breeding. Artificial light is provided in the growth chamber by using either LED lights, halogen lamps, high pressure sodium (HPS) lights, or metal halide (MH) bulbs that give photo-synthetically active radiation (PAR) to the plants for photosynthesis. The temperature and humidity vary for each crop [173].

Usually, 60–70% humidity is ideal for plants. The photoperiod usually varies for each crop but the basic requirement is around 22 h of photoperiod with 2 h of darkness [174]. Light duration and intensity increase the development rate for long-day crops but inhibit development in short-day plants, and plants take it as a stress condition by showing adverse effects, developing leaf injuries and no flowering. Thus, giving low supplementary light is found to be more effective than providing high intensity light. Lights having low photo-synthetically active photons (PPF) were found to be more beneficial for plants and are also considered to be more cost effective [175]. 

### 6.1. An Amalgamation of Speed Breeding with GETs

**Figure 7:**
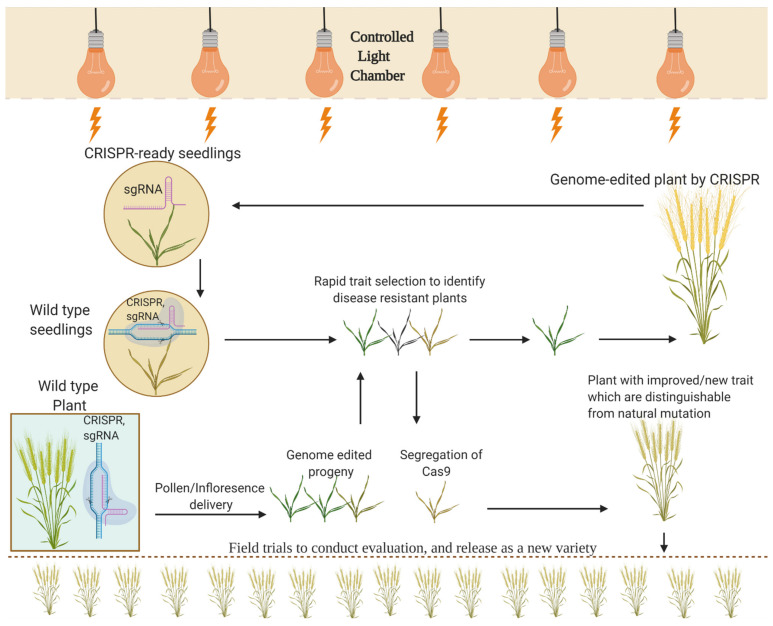
Speed breeding coupled with GETs, which is called the ExpressEdit approach, to shorten the generation time can be used directly in speed breeding process. To save time, Cas9 gene and sgRNA sequences can be applied directly to plants without regenerating the plants in labs. During the rapid trait selection (screening), segregated progenies are screened and isolated for the new trait (such as disease resistance), and also identify the plants that do not contain Cas9 but have new traits. Alternatively, CRISPR-ready plants may contain Cas9 and can be subjected for the further cycles of gene editing. However, all the selected plants undergo field trials and strict evaluation to release a new variety (shown in the below side of figure).

Speed breeding can save time in generation development by amalgamating it with GETs. GETs can only edit one or two non-elite genotypes, which are passed through a complex process of transformation and tissue culturing to regenerate the edited plants. Due to the latest innovations, GETs can edit the elite genotypes with increased transformation efficiency [176,177]. Despite a massive improvement in GE technology, this still requires a complex tissue culturing process and gRNA designing, and Cas9 protein recruitment, which cannot be performed without molecular lab work [178]. A new method, “ExpressEdit”, (Figure 7) that is carried out by coupling the speed marker-assisted selection and preassembled GETs components (sgRNA and Cas proteins) with the speed breeding technique, which can exclude the sensitive callus culturing step [18]. GETs’ components such as sgRNA and Cas9 protein can be delivered into plant cells (mature seeds, plant shoot apical meristems) by employing the Gemini viruses vectors or direct particle bombardment, for example, in wheat [179,180]. The plant, having a Cas9 gene, is called CRISPR ready. Furthermore, the segregating progeny is screened to identify the plants that have new traits with or without the Cas9 gene. The plants having the Cas9 gene can be further subjected to editing with the use of sgRNA to target different genes. In a nutshell, speed breeding with other additions as stated above can keep the pace of a crop’s improvement in the face of persistent global challenges such as the ever-increasing population and low food production [18].

### 6.2. Achievements of Speed Breeding

In wheat, multiple traits are related to diseases such as leaf rust and root architecture that were highly variable in both the field and in greenhouses. The required high throughput repeatable methods for screening were easily achieved by speed breeding. It is cost-efficient and took less time in screening as compared to conventional breeding. This robust method allows screening of the germplasm more rapidly, thus proving highly efficient for variable traits [166,169]. Improvements in wheat plant height, flowering period, and resistance to several diseases can be achieved by speed breeding [169]. 

In Argentina, Scarlett is the most extensively cultivated cultivar of barley, which is susceptible to different diseases. By taking four lines with modified backcrossing methodology, resistant lines were developed within 2 years that were disease resistant and yielded more than the cultivar [165]. In barley, the glaucousness on the leaf sheath is an important trait for the plant to survive under hot climatic conditions [181]. This drought-tolerant trait in barley can be obtained by speed breeding [182]. 

Rice is the most sensitive cereal crop to salt stress. Breeding to obtain salt-tolerant varieties takes many years, which makes the task difficult. With the use of advanced techniques such as SNP and whole-genome sequencing, it becomes easier for the breeders to insert genes and then achieve several generations each year with the help of speed breeding [183]. In rice, a new salt-tolerant line “YNU31-2-4” was developed with the help of speed breeding. After inserting genes by SNP, the breeding cycle was accelerated with the help of speed breeding methodology by using optimum light, 14 h light and 10 h darkness, from germination to the 30th day to allow the plant to complete its vegetative phases and, after this, 10 h light and 14 h darkness were provided to initiate the reproductive phase. The tillers were removed, and the embryo rescue technique was used to save time before seed maturity. Thus enabling the researchers to obtain four to five generations of rice per year (Table 3) [184].

**Figure 8:**
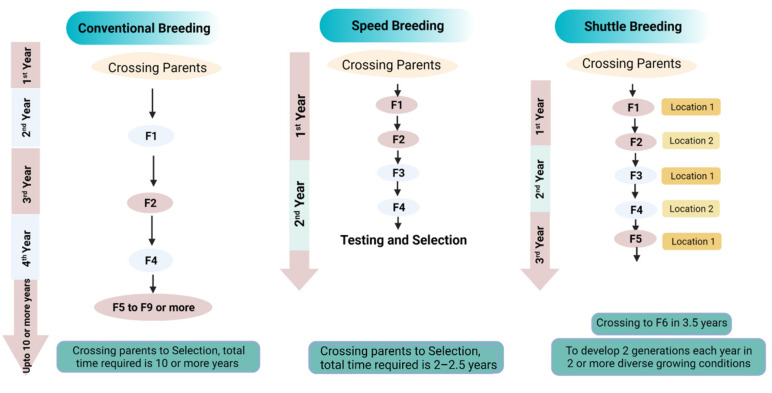
Illustrates the schematic flow of conventional breeding, speed breeding, and shuttle breeding regarding the requisite time of each method.

A considerable improvement in breeding has been achieved as compared to double haploid (DH) technology, which faces several agronomic drawbacks: low germination rate, poor vigor, and sometimes distorted growth [187]. Moreover, developing DH lines is costlier as compared to speed breeding, which does not require any specific precision [188]. Following specific protocols in speed breeding has enhanced the efficiency of this method and has surpassed most of the drawbacks DH technology faces. The time required in developing DH lines is more as compared to speed breeding. The poor seed germination in DH technology is overcome by using a specific light intensity and duration protocol. Different light regimes and temperatures are adjusted, which increases the germination rate [172]. Moreover, treating seeds with gibberellin has also increased the germination percentage in speed breeding [18]. Speed breeding with single seed descent (SSD) is useful to screen for diverse germplasm within a short time by hastening the breeding cycles [16]. SSD with speed breeding is time-saving and cost-efficient as compared to the conventional pedigree breeding method [167]. Shuttle breeding is another approach used to grow crops in diverse ecological conditions. The methodology was successful in developing resistant crop varieties with more yield and adaptability to various conditions. Speed breeding surpasses this methodology too and produces a three times greater number of generations. With shuttle breeding, only two generations per year can be achieved, while with speed breeding up to six generations can be obtained (Figure 8) [189].

However, despite the potential advantages of the speed breeding program, it still faces limitations in its adoption due to the lack of technicians and trained breeders for this method. The proper regulation of the methodology requires the control of temperature, photoperiod, and other factors, but due to the lack of infrastructure, this method is limited to a small scale only and is not practiced on a commercial level. In developing countries, this method is still waiting for governments to approve it and give financial assistance for its adoption.

Speed breeding in upcoming years will have a revolutionary impact on the breeding strategies to overcome the challenges faced by breeders, especially the generation time. Speed breeding, coupled with other biotechnological techniques, can further accelerate the generation cycle. It can be coupled with CRISPR/Cas9- or RNP-mediated genome editing to knock out the genes that prolong the vegetative period and induce late flowering or are insensitive to photoperiodism. Knocking out these genes could be helpful in further reducing generation time. Moreover, breeders are taking necessary measures to reduce the cost and make it more efficient so that it can be adopted by developing countries too.

## 7. Future Prospects

Before the advent of CRISPR/Cas9, conventional breeding was the major approach used towards the improvement of agronomic traits by exploiting the genetic variation that is caused by mutagens and cross-breeding. In the present era, CRISPR/Cas9 is time efficient and has improved GET, as it inserts and deletes the DNA at specified genomic sites, either by the NHEJ or HDR repairing mechanism. Despite CRISPR/Cas9’s importance, it is associated with limitations, including off-target effects, and can cut only a single targeted genomic site. The discovery of CRISPR/Cpf1 has played its multiplayer role by targeting multiple genomic sites to improve the crops. Along with CRISPR/Cas9, CRISPR/Cpf1 is also prone to off-target effects, which could lead to undesirable traits. 

### 7.1. Solutions by Improved GETs

The development of dCas9 protein has solved the problems of off-target effects up to a maximum extent by only requiring the gRNA to modify the single nucleotide bases at targeted genomic sites. As an example, base editing, prime editing, and dCas9-based epigenetic modifications work by amalgamating gRNA with dCas9 protein, which is responsible for point mutations and increasing the efficiency of the GETs. Crops that are improved by GE approaches are considered transgenic and face strict GMO regulatory rules. These GMO concerns could be ruled out by isolating the transgene-free segregants in the next generations, after the development of transgenic plants by the above-stated method in the “transgene-free development” section. However, not all GMO plants are transgene, as some genes are silenced or deleted in an organism for some desirable results. The varieties possessing silenced genes are genetically modified but are not transgene, so transgenic plants are GMOs, but not all GMOs are transgenic plants. 

### 7.2. Increase in Regeneration Efficiency 

Indeed, the novel GETs have improved the production of cereal crops. However, these methods necessitate the work of vector construction, which could lead to reduced plant transformation due to regeneration and transformation inefficiency. 

Before the introduction of developmental regulators, the tissue culturing technique was used by exploiting the totipotency mechanism of plants and generating somatic cells, which afterward develop into specific cell types. However, this method takes a lot of time and the hormonal requirements for each specific plant species, race, or cultivar, which complicates the development of a healthy plant. Using developmental regulators can compensate the time required for transformation by inducing edited somatic cells to form meristems, thus narrowing the whole procedure. The seedlings are treated with the *Agrobacterium* strain possessing gene-edited reagents with developmental regulators, which induce meristems growth from edited plant somatic cells. The meristems that develop into a shoot are then removed and only roots are induced. This technique is newly developed and has shown tremendous results in *Arabidopsis* but requires more attention for its improvement in other plant species such as cereals. 

### 7.3. Speed Breeding to Rule out GMOs and Shorten the Breeding Procedure

The speed breeding-based approach utilizes continuous light to shorten the generation time, and it can be employed by researchers to increase the breeding speed. Moreover, speed breeding not only develops 3–4 crops’ generations in a year but also rules out the GMOs’ restrictions by adapting conventional breeding methods. Despite a lot of modifications in already existing molecular and conventional breeding methods, it is in dire need of further improvement to the available breeding approaches in pursuance of the ever-increasing food demand. Furthermore, this speed breeding method can be coupled with GETs such as the ExpressEdit approach to shorten the generation development time by reducing the wet-lab work However, some disadvantages are associated with speed breeding such as optimization of the speed breeding protocol, temperature-sensitive crops, accessibility of suitable facilities, and trained staff. 

### 7.4. Transcriptional Factors

**Figure 9:**
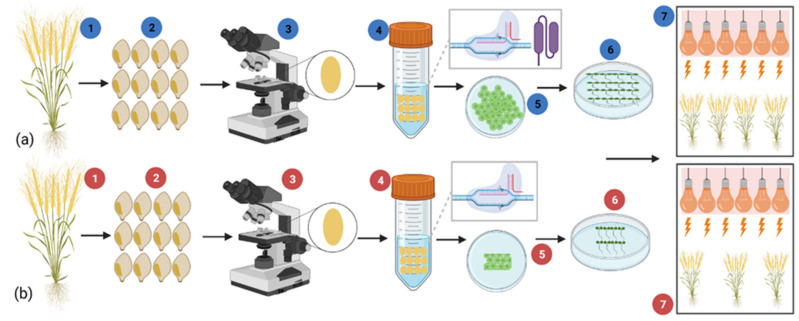
(**a**,**b**) show the CRISPR/Cas9 *Agrobacterium* transformation of wheat by integrating it with speed breeding to reduce the generation time. Blue and red color shows the transformation with GRF–GIF and without it, respectively. This procedure consists of different steps: (1,2) Screening of best lines and collection of germplasm, (3) embryo collection under a microscope, (4) vector construction and *Agrobacterium* transformation (blue with GRF–GIF and red without it), (5) Auxin callus induction, more in (**a**,**b**), (6) antibiotic selection (do not need in GRF–GIF method), (7) integration of speed breeding with the CRISPR/Cas9.

Moreover, GETs may cause lower transformation efficiency in cereal crops such as wheat. However, these associated problems can be solved by using a recently developed method, GRF–GIF (Figure 9). Growth Regulatory Factors (GRFs) are the specific kind of transcriptional factors that work by interacting and forming a transcriptional complex with Growth Interacting Factors (GIFs) [190].

These factors play a pivotal role in the growth promotion of a plant’s lateral parts with reproductive competence, and any mutation in these factors directly affects the size, shape, and functionality of those parts. Several regulatory and interacting factors (GRF–GIF) are identified in plants, but the transcriptional complex formed by “GRF4–GIF1” showed robust growth due to their homology [191]. Both wheat and rice plants showed an increase in regeneration efficiencies with desirable results in terms of plant fertility and development. Hence, it can be utilized in those plants with a slower growth mechanism [192]. Combining genome editing techniques with these regulatory factors and speed breeding can have a revolutionary impact on the history of genome editing to increase the production of cereals.

## Figures and Tables

**Table 1:** Comparisons between different GETs.

Functions	EMNs	ZFNs	TALENs	CRISPR/Cas9	Base Editing	CRISPR/Cpf1	References
Mode of action	In the target region direct conversion of information stand	In the target region double-strand breaks	In the targeted DNA region double-strand breaks	In the targetedDNA region double-strands or single-strand breaks	Single-Stranded Base changing	Double-stranded breaks	[19,20]
Target recognition	Good	Good	Good	Good	Very Good	Very Good	[20,21]
Mutation rate	Average	High	Average	Low	Low	High	[19,20,22,23]
Creation of large-scale libraries	Difficult to do	Impossible	Difficult to do	Possible	Possible	Possible	[20,24,25]
Multiplexing	Technically difficult	Hard to do	Hard to do	Possible	Possible	Possible	[19,20,26,27]
Components	Exogenous polynucleotide (chimeraplast)	Zn finger domains Nonspecific FokI nuclease domain	TALE DNA-binding domains Nonspecific FokI nuclease domain	Cas9 proteins, crRNA	CBEs, ABEs	Cpf1 proteins, crRNA	[20,21,28,29]
Structural protein	Dimeric protein	Dimeric protein	Dimeric protein	Monomeric Protein	Monomeric Protein	Monomeric Protein	[19,23]
Catalytic Domain	Absence of a catalytic domain	Restriction endonuclease FokI	Restriction endonuclease FokI	RuvC and HNH		RuvC and HNH	[20,28]
Length of the target sequence (bp)	68–88	24–36	24–59	20–22	Point Mutation	20–24	[23,24,29,30]
Protein engineeringsteps	Not required	Required	Required	Not difficult to testgRNA		Not difficult to testgRNA	[20,25,31]
Cloning	Unnecessary	Necessary	Necessary	Unnecessary		Unnecessary	[20,25,31]
gRNA production	Not essential	Cannot apply	Cannot apply	Can produce easily		Can produce easily	[20,25,32]
Target GETs	Not essential	ZFN Genome v2.0 ZifBASE Zinc-Finger Database (ZiFDB) Zinc-Finger Tool EENdb	TALE-NT 2.0 SPATA TALEN offer TALEN Library	CHOP CHOP CRISPRs web Server Crass: The CRISPRAssembler CRISPR Target	Cas nickase, Cpf1 adenosine deaminases, Cas13b	Breaking-Cas Cas-OFFinder CRISPORCCTOP	[29,31,33]
Off-target effects	Low off-target effect	Low off-target effect	Shows least off-target activities	Low off-target effect	Very Low	Low off-target effect	[20,22]
Cost of development	High	High	Higher	Low	Low	Low	[20,24,25]

**Table 2:** Achievements in cereals by using GETs.

Gene Editing Tool	Crop	Targeted Gene	Targeted Trait	Reference
CRISPR/Cas9	Wheat	*TaLOX2*	Development of grain	[72]
CRISPR/Cas9	Maize	*LIG1, Ms26. Ms45, ALS1,* and *ALS2*	Chlorsulfuron-resistant	[73]
CRISPR/Cas9	Rice	*GS3, GW2, GW5, TGW6,*	Improved grain related parameters	[74]
CRISPR/Cas9	Wheat	*Gli-2 loci*	Low-gluten foodstuff	[75]
CRISPR/Cas9	Rice	*OsPRX2*	Improved salt tolerance level	[76]
CRISPR/Cas9	Wheat	*TaInox, TaPds*	Chlorophyll synthesis	[27]
CRISPR/Cas9	Rice	*Waxy*	Enhanced glutinosity	[77]
CRISPR/Cas9	Rice	*Hd2, Hd4, Hd5*	Early heading	[78]
CRISPR/Cas9	Maize	*PPR, RPL*	Reduced zein protein	[79]
CRISPR/Cas9	Maize	*ARGOS8*	Drought tolerance	[80]
CRISPR/Cas9	Rice	*OsNAC041*	Salt tolerant	[81]
CRISPR/Cas9	Maize	*ZmHKT1*	Salt tolerant	[82]
CRISPR/Cas9	Rice	*LAZY1*	Tiller spreading	[83]
CRISPR/Cas9	Rice	*Gn1a, GS3, DEP1*	Enhanced grain number, larger grain size, and dense erect panicles	[84]
CRISPR/Cas9	Wheat	*GW2*	Increased grain weight and protein content	[85]
CRISPR/Cas9	Wheat	*TaGASR7, TaGW2, TaDEP1, TdGASR7(durum wheat)*	Grain development, kernel length, storability, and plant height and weight	[86]
CRISPR/Cas9	Wheat	*TaGW2, TaGASR7*	Grain and kernel length and weight	[87]
CRISPR/Cas9	Wheat	*α-gliadin, gamma-gliadins*	Gliadins	[88]
CRISPR/Cas9	Wheat	*TaLOX2, TaUbil1*	Grain development	[89]
CRISPR/Cas9	Wheat	*TaDREB2,* *TaERF3*	Drought signaling	[90]
CRISPR/Cas9	Wheat	*TaCER9, TaLOX2,* *TaGW2*	Grain development	[91]
CRISPR/Cas9	Wheat	*TaGW2, TaLpx-1, TaMLO*	Kernel width and weight; resistance to powdery mildew	[92]
CRISPR/Cas9	Wheat	*α-gliadin genes*	Low-gluten wheat	[75]
CRISPR/Cas9	Wheat	*TaMs45*	Male fertility	[93]
CRISPR/Cas9	Rice	*OsSWEET13*	Bacterial blight resistance	[94]
CRISPR/Cas9	Rice	*SBEIIb*	High amylose content	[95]
CRISPR/Cas9	Wheat	*EDR1*	Powdery mildew resistance	[96]
CRISPR/Cas9	Rice	*OsERF922*	Enhanced rice blast resistance	[97]
CRISPR/Cas9	Rice	*OsSWEET13*	Bacterial blight resistance	[97]
CRISPR/Cas9	Maize	*TMS5*	Thermosensitive male-sterile	[98]
CRISPR/Cas9	Rice	*OsMATL*	Induction of haploid plants	[99]
CRISPR/Cas9	Rice	*OsPIN5b and GS3,* *OsMYB30*	High yielding and cold tolerance	[100]
CRISPR/Cas9	Rice	*ALS*	Herbicide resistance	[72]
CRISPR/Cas9	Rice	*LAZY1*	Tiller spreading phenotype	[83]
CRISPR/Cas9	Rice	*Gn1a,* *DEP1, GS3*	Number of grains, erect panicles, specific for grain size	[84]
CRISPR/Cas9	Rice	*SBEIIb*	High amylose rice	[95]
CRISPR/Cas9	Rice	*OsERF922*	Rice blast resistance	[94]
CRISPR/Cas9	Rice	*OsEPSPS*	Glyphosate resistant	[101]
CRISPR/Cas9	Rice	*ALS*	Herbicide resistance	[99]
CRISPR/Cas9	Rice	*ALS*	Herbicide resistance	[102]
CRISPR/Cas9	Rice	*EPSPS*	Herbicide resistance	[101]
CRISPR/Cas9	Rice	*ALS*	Herbicide resistance	[103]
CRISPR/Cas9	Maize	*ALS*	Herbicide resistance	[73]
CRISPR/Cas9	Maize	*ARGOS8*	Drought stress tolerance	[104]
CRISPR/Cas9	Wheat	*TaMLOA1, TaMLOB1,* *TaMLOD1*	Resistance to powderyMildew	[105]
CRISPR/Cas9	Maize	*PDS, IPK1A, IPK*	Phytic acid content	[106]
CRISPR/Cpf1	Rice	*OsEPFL9*	To regulate the stomatal density in leaf	[107]
CRISPR/Cpf1	Rice	*OsROC5* and *OsDEP1*	Editing efficiency was compared on varying temperature	[86,87]
CRISPR/Cpf1	Maize	*GL2*	Editing efficiency was compared on varying temperature	[108]
CRISPR/Cpf1	Rice	*DL, ALS, NCED1, AO1*	Drooping leaf phenotype	[32]
CRISPR/Cpf1	Rice	*OsPDS, OsBEL*	Heritable mutations	[109,110]
CRISPR/Cpf1	Rice	*OsRLK, OsBEL*	Albino phenotype	[111]
CRISPR/Cpf1	Maize	*glossy2*	Efficiency compared with CRISPR/Cas9	[112]
CRISPR/Cpf1	Rice	*OsPDS, OsGS3*	Improved the editing efficiency	[113]
CRISPR/Cpf1	Rice	*OsDEP1, OsROC5, OsPDS*	Tenfold reduction in miR159b transcription, transcriptional repression	[114]
CRISPR/Cpf1	Rice	*DEP1, PDS, EPFL9*	Efficient editing at all TTTV PAM sites	[115]
TALENs	Rice	*OsSWEET14*	Bacterial blight resistance	[116]
TALENs	Wheat	*TaMLO*	Powdery mildew resistance	[105]
TALENs	Maize	*ZmGL2*	Reduced epicuticular wax in leaves	[117]
TALENs	Rice	*OsBADH2*	Fragrant rice	[118]
TALENs	Rice	*DEP1, CKX2, BADH2, SD1*	Rapid and efficient gene modification in rice	[119]
TALENs	Maize	*ZmMTL*	Induction of haploid plants	[120]
TALENs	Maize	*PDS, IPK1A, IPK* and *MRP4*	Reduce the phosphorous concentration	[121]
TALEN	Wheat	*TaMLO*	Powdery mildew resistance	[105]
ZFN	Maize	*PAT*	Herbicide resistance	[122]
ZFN	Rice	*OsQQR*	Detection of safe harbor loci herbicide	[123]
ZFNs	Maize	*ZmIPK1*	Herbicide tolerant and phytate reduced maize	[124]
ZFNs	Maize	*ZmTLP*	Trait stacking	[125]
ZFNs	Rice	*OsQQR*	Trait stacking	[123]
MNs	Maize	*lg1,* *ms26*	Targeted mutation	[126]
MNs	Maize	*ms26*	Male sterility	[127]
MNs	Wheat	*DsRed*	Removed selectable markers	[128]

**Table 3:** Traits improved using speed breeding technique.

Species	Trait/Diseases Improved	No. of Generations per Year	References
Spring Wheat	Stripe rust, seminal root number and angel, and plant height.	4–6 generations per year	[16]
	Yellow spot		[169]
	Black rust		[169]
	High protein rate andtolerant to preharvest sprouting		[185]
	Brown rust		[186]
	Stem rust		
Durum Wheat	Crown rust	Up to 6 generations per year	[166]
Canola	Pod shattering	4 generations per year	[16]
Barley	Net form net blotch	4–6 generations per year	[165]
	Spot form net blotch		
	Spot blotch		
	Leaf rust		
	Glaucousness drought-tolerant trait		[16]
Rice	Salt tolerance	4 generations per year	[184]
